# Pyrimethamine Modulates Interplay between Apoptosis and Autophagy in Chronic Myelogenous Leukemia Cells

**DOI:** 10.3390/ijms22158147

**Published:** 2021-07-29

**Authors:** Young Yun Jung, Chulwon Kim, In Jin Ha, Seok-Geun Lee, Junhee Lee, Jae-Young Um, Kwang Seok Ahn

**Affiliations:** 1 Department of Science in Korean Medicine, Kyung Hee University, 24 Kyungheedae-ro, Dongdaemun-gu, Seoul 02447, Korea; ve449@naver.com (Y.Y.J.); sunny10526@nate.com (C.K.); seokgeun@khu.ac.kr (S.-G.L.); jyum@khu.ac.kr (J.-Y.U.); 2 Korean Medicine Clinical Trial Center (K-CTC), Korean Medicine Hospital, Kyung Hee University, Seoul 02447, Korea; ijha0@naver.com (I.J.H.); ssljh@khu.ac.kr (J.L.)

**Keywords:** Pyrimethamine, STAT5, apoptosis, autophagy, CML

## Abstract

Pyrimethamine (Pyri) is being used in combination with other medications to treat serious parasitic infections of the body, brain, or eye and to also reduce toxoplasmosis infection in the patients with HIV infection. Additionally, Pyri can display significant anti-cancer potential in different tumor models, but the possible mode of its actions remains unclear. Hence, in this study, the possible anti-tumoral impact of Pyri on human chronic myeloid leukemia (CML) was deciphered. Pyri inhibited cell growth in various types of tumor cells and exhibited a marked inhibitory action on CML cells. In addition to apoptosis, Pyri also triggered sustained autophagy. Targeted inhibition of autophagy sensitized the tumor cells to Pyri-induced apoptotic cell death. Moreover, the activation of signal transducer and activator of transcription 5 (STAT5) and its downstream target gene Bcl-2 was attenuated by Pyri. Accordingly, small interfering RNA (siRNA)-mediated STAT5 knockdown augmented Pyri-induced autophagy and apoptosis and promoted the suppressive action of Pyri on cell viability. Moreover, ectopic overexpression of Bcl-2 protected the cells from Pyri-mediated autophagy and apoptosis. Overall, the data indicated that the attenuation of STAT5-Bcl-2 cascade by Pyri can regulate its growth inhibitory properties by simultaneously targeting both apoptosis and autophagy cell death mechanism(s).

## 1. Introduction

Pyrimethamine [Pyri] (2,4-diamino-5-p-chlorophenyl-6-ethyl-pyrimidine), which belongs to the antifolate class of drugs, has been used as an anti-parasitic medication for the treatment of toxoplasmosis [1]. It can effectively block the enzyme dihydrofolate reductase to mitigate the folic acid synthesis for DNA synthesis of cofactor. Pyri has also been used to treat infections occurred by protozoan parasites like *Toxoplasma gondii* and *Plasmodium falciparum* [2]. Additionally, Pyri can exhibit immunomodulatory activities and promote apoptosis through causing upstream activation of caspases and Bcl-2 suppression in blood lymphocytes [3,4,5,6,7]. 

Chronic myelogenous leukemia (CML) is a kind of clonal disease linked with the presence of Philadelphia chromosome (Ph), which is generated by 9;12 chromosomes reciprocal translocation, and it can cause aberrant activation of oncogenic fusion kinase Bcr-abl [8,9,10,11,12]. Bcr-abl has been also reported to drive the abnormal proliferation of cancer cells through the constitutive activation of multiple oncogenic signaling pathways such as Mitogen-activated protein kinase kinase/ extracellular-signal-regulated kinase (MEK/ERK), PI3K, and Janus kinase/Signal transducer and activator of transcription (JAK/STAT) signaling pathways [13,14,15,16,17]. The advent of several tyrosine kinase inhibitors (TKIs) have successfully controlled the progression of CML. However, TKI-associated drug resistance and potential of relapse during drug discontinuation has prompted the discovery of novel agents with lower toxicities and better therapeutic efficacy [18]. A number of studies have indicated that the drugs obtained from Mother Nature can effectively function as novel anti-cancer therapies with lower drug resistance [19,20,21,22,23].

Apoptosis is one of the major representative forms of physiological cell death mechanisms [24,25,26]. It can be distinguished from necrosis because the latter occurs by accidental and acute damage to cells [27,28]. In contrast, apoptosis has been reported to be a strictly controlled phenomenon by hierarchical molecular signatures belonging to *Caenorhabditis elegans*, later observed to be conserved in the mammalian cells [29,30,31]. Proteolytic cleavage of poly (ADP-ribose) polymerase (PARP), a nuclear enzyme that can control DNA stability, DNA repair, and transcriptional regulation is another hallmark feature of apoptosis. In addition, caspase can cleave the 116-kDa of PARP to 85- and a 24-kDa fragment for activation [32,33,34,35,36,37]. Therefore, several studies have used both PARP and caspases as apoptosis markers [26,38,39].

Autophagy is an essential mechanism for maintaining cellular homeostasis [40,41]. The autophagy process can effectively remove the misfolded and damaged proteins which can drive, starvation, tumor development and suppression [42,43]. Autophagy is kind of intracellular degradative process, and it can be initiated in the presence of poor nutritional and hypoxia conditions or upon exposure of cancer cells to chemotherapy [44,45,46]. Interestingly, Bellodi et al. reported that pharmacological inhibition of autophagy can induce cellular apoptosis. When autophagy-related proteins such as ATG7 and ATG5 were knocked down. However, an opposite active trend was observed in apoptosis in CML [47]. Overall, both autophagy and apoptosis have been reported to play an important role in protection against cellular damage and development of cancer. 

Signal transducer and activator of transcription 5 (STAT5) has been known to play a significant role in regulating cellular survival and homeostasis. However, its aberrant activation has been reported to mediate tumorigenesis [48,49]. The different STAT family members [50,51,52,53], including STAT5, can promote the development of various cancers such as solid tumors and hematological malignancies [54]. Activation of the STAT5 signaling pathway is initiated upon its phosphorylation by intracellular kinases like JAK1, JAK2, and Src [55,56,57,58]. Additionally, previous studies have suggested that pharmacological inhibition of STAT5 phosphorylation could induce the programmed cell death mechanism(s) like autophagy and apoptosis in human lung and mesangial cancer cells [39,59]. 

In this study, we focused on the effect of Pyri in modulating autophagy and apoptosis by acting upon different programmed cell death processes. We also noted that the Pyri-induced STAT5 inhibition could effectively mediate autophagy and apoptosis activation in CML cells. 

## 2. Results

### 2.1. Pyri Reduced the Viability of Human Myelogenous Leukemia Cell Lines

**Figure 1:**
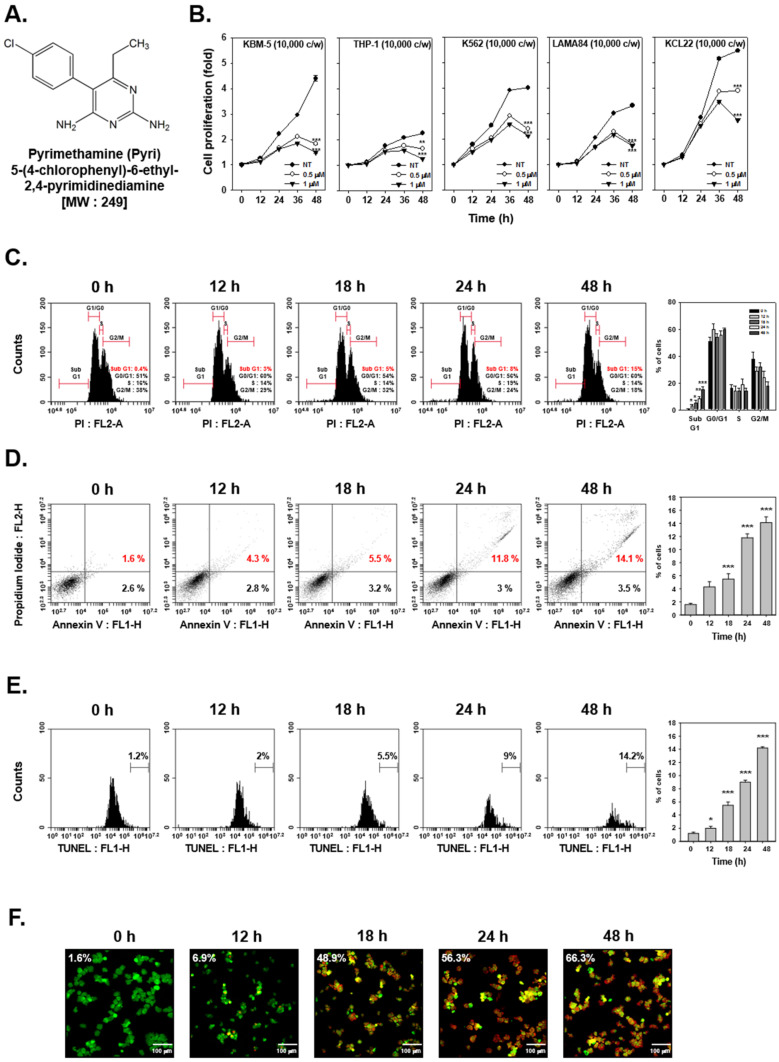
Pyri induces apoptosis and suppresses proliferation in CML cells. (**A**) The chemical structure of Pyrimethamine (Pyri). (**B**) KBM5, THP-1, K562, LAMA84, and KCL22 cells (1 × 10^4^ cells/well) were seeded onto 96-well plates, they were left non-treated (NT, ●), treated with Pyri at 0.5 μM (○), and 1 μM (▾) for the indicated time intervals. The cell viability was measured using MTT assay. (**C**–**E**) KBM5 cells (1 × 10^6^ cells/well) were seeded onto 6-well plates, incubated at 37 °C with 1 μM of Pyri for various time intervals. Thereafter, the cells were fixed and analyzed using flow cytometry for cell cycle analysis, Annexin V, and TUNEL assays. *** *p* < 0.001 vs. non-treated (NT) cells, ** *p* < 0.01 vs. non-treated (NT) cells, * *p* < 0.05 vs. non-treated (NT) cells. (**F**) The cells were treated with 1 μM of Pyri for 0, 12, 18, 24, 48 h. The cells were stained with a Live/Dead assay reagent for 30 min and then analyzed under a fluorescence microscope (scale bar: 100 µm) as described in “Materials and methods.” Percentage indicates dead cells (red). The results shown are representative of three independent experiments.

First, we determined whether Pyrimethamine (Pyri) (Figure 1A) can influence the viability of human myelogenous leukemia KBM5, THP-1, K562, LAMA84, and KCL22 cell lines. All cell lines were treated with Pyri (0, 1, 0.5 µM) for indicated time intervals and viability was measured by MTT assay. It was noted that Pyri significantly suppressed the cell viability but was especially effective against KBM5 cells as compared with non-treated control (Figure 1B). 

### 2.2. Pyri Stimulated the Apoptosis-Induced Cell Death

To confirm the effects of Pyri on apoptotic cell death, cell cycle analysis, Annexin V and TUNEL assays were conducted. As indicated in Figure 1C, cell cycle distribution was analyzed with PI-stained cells and the results showed that sub G1 phase arrest was observed in Pyri treated cells in a time-dependent fashion. Next, cells were probed with Annexin V-FITC and analyzed by flow cytometer. Annexin V assay can sort the cells into necrosis and apoptotic cells as shown on Figure 1D, Pyri induced apoptosis with increasing time intervals. Thereafter Pyri-treated cells were stained with TUNEL, and apoptotic cells were probed after TUNEL staining. TUNEL probed cells showed peak movement, and the results suggested that the peaks of the Pyri-treated cells shifted effectively to the right with increasing percentage (Figure 1E). Next, cell death caused by Pyri was investigated by live and dead assay. A significant increase in the percentage of dead cells was noted upon treatment with Pyri at different time intervals and drug doses (Figure 1F).

### 2.3. Pyri Suppressed the Protein and mRNA Expression of Anti-Apoptotic and Oncogenic Markers

**Figure 2:**
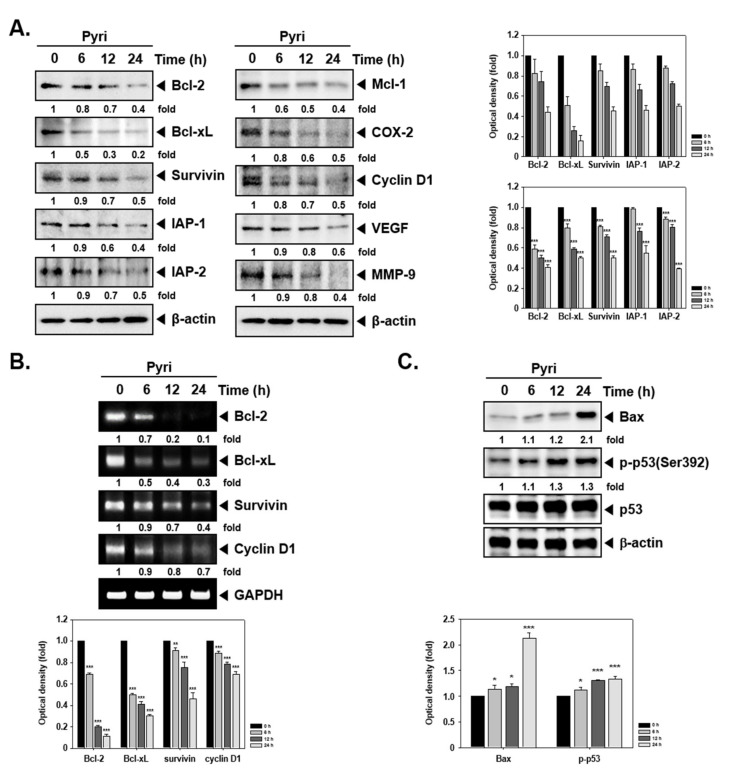

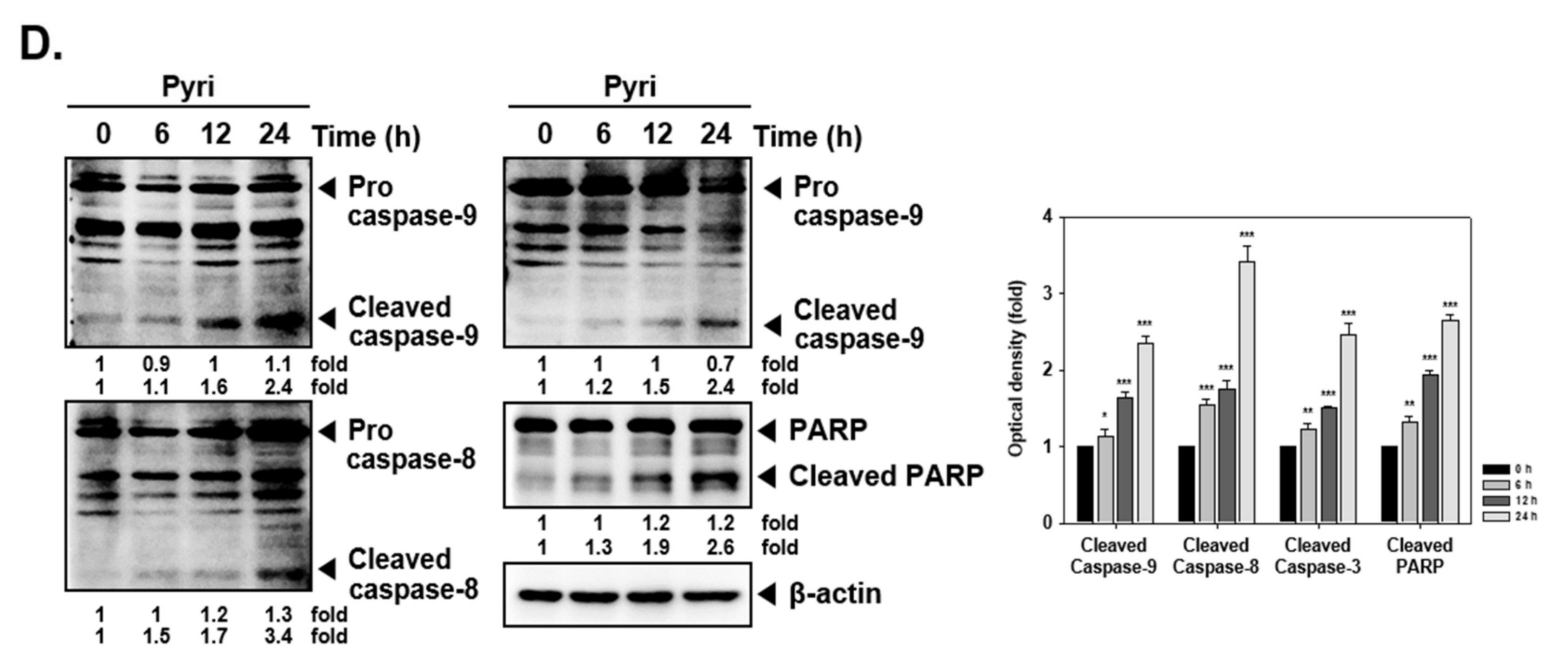
Pyri downregulates the expression of various tumorigenic proteins. (**A**) KBM5 cells (1 × 10^6^ cells/well) were treated with 1 µM of Pyri for indicated time intervals and western blotting was performed (**B**) KBM5 cells were treated with Pyri (1 µM) as described above and RT-PCR analysis was done. (**C**,**D**) KBM5 cells were treated with 1 µM of Pyri for indicated time intervals, and western blotting was carried out. *** *p* < 0.001 vs. non-treated (NT) cells, ** *p* < 0.01 vs. non-treated (NT) cells * *p* < 0.05 vs. non-treated (NT) cells. The results shown are representative of three independent experiments.

To evaluate the mechanism(s) regulating apoptosis, the expression of anti-apoptotic and oncogenic proteins by Western blot analysis was studied. Pyri treated cells displayed a substantial decrease in the level of various proteins such as (Bcl-2, Bcl-xl, Mcl-1, Survivin, IAP-1, IAP-2, COX-2, Cyclin D1, VEGF, and MMP-9) in a time-dependent fashion (Figure 2A). The expression of Bcl-2, Bcl-xl, Survivin, and Cyclin D1 was also evaluated by RT-PCR, and the results indicated that Pyri can effectively suppress mRNA expression levels of these markers as well (Figure 2B). 

### 2.4. Pyri Induced Apoptosis through PARP and Caspase Cleavage

The expression of Bax and p53 was next evaluated in Pyri-treated KBM5 cells. These proteins can regulate apoptotic cell death by diverse mechanisms. For instance, Bax can induce apoptosis, whereas p53 has been related to cell death and can induce the cell cycle arrest. As depicted in Figure 2C, pyri induced Bax and p-p53 expression but did not affect the expression of p53 protein. We also investigated the action of Pyri on PARP and caspase cleavage by Western blot analysis. It was noted that depending upon time intervals, caspase-9, 8, 3, and PARP cleavage was increased (Figure 2D). The untagged bands shown in the blot are non-specific bands.

### 2.5. Pyri Increased the Autophagy-Induced Cell Death in KBM5 Cells 

**Figure 3:**
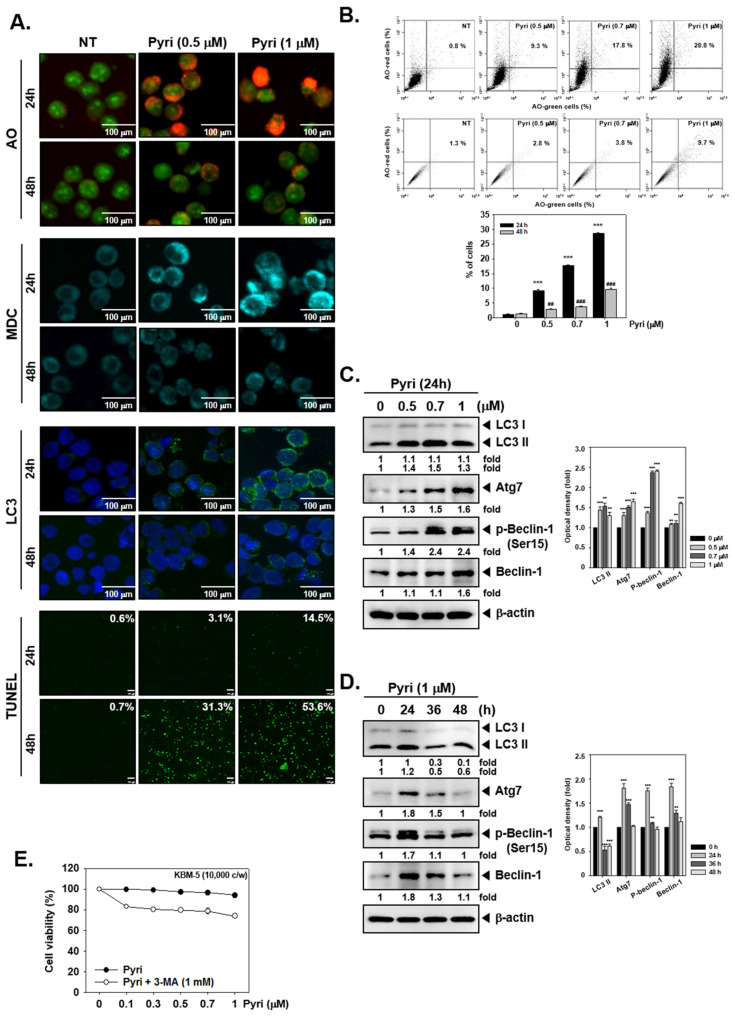
Pyri triggers substantial autophagy. (**A**) Representative images of Acridine orange (AO) (*first*) and Monodansylcadaverine (MDC) (*second*) staining of KBM5 cells following treatment with Pyri at the indicated concentration for the indicated time. For AO staining (*first*), red color intensity showed acidic vesicular organelles, representing the formation of autophagolysosomes. For MDC staining (*second*), punctate fluorescence in the cytoplasm indicates the formation of autophagic vacuoles. Representative images of LC3 (*third*) immunocytochemistry of KBM5 cells following treatment with Pyri. Representative images of TUNEL staining of KBM5 cells (*fourth*) after treatment with the indicated concentration of Pyri for the indicated time. (scale bar: 100 µm) (**B**) KBM5 cells were treated with the indicated concentration of Pyri for 24 and 48 h, then cells were stained with AO. Cell autophagy was analyzed by quantification of acidic vesicular organelles (AVOs) with AO using flow cytometry. *** *p* < 0.001 vs. non-treated (NT) cells, ** *p* < 0.01 vs. non-treated (NT) cells for 24 h, and ### *p* < 0.001 vs. non-treated (NT) cells, ## *p* < 0.01 vs. non-treated (NT) cells for 48 h. (**C**) KBM5 cells (1 × 10^6^ cells/well) were treated with indicated concentration of Pyri for 24 h, and whole-cell extracts were subjected to western blotting (**D**) KBM5 cells (1 × 10^6^ cells/well) were treated with 1 µM of Pyri for indicated time intervals, and whole-cell extracts were subjected to western blot analysis for various proteins (**E**) Inhibition of autophagy with 3-MA decreased the viability of Pyri treated cells. Cell viability was measured by MTT assay after cells were incubated with the indicated concentration of Pyri with 3-MA (±3-MA, 1 mM) for 24 h. The results shown are representative of three independent experiments.

We next investigated the effects of Pyri on autophagy activation in KBM5 cells. As autophagy produces acidic vesicular organelles [60], the autophagy activation was monitored through acidic components staining by Monodansylcadaverine (MDC) or acridine orange (AO). As shown in results, Pyri treatments affected the production of acidic components, especially the expression was substantially induced in 1 µM of Pyri treated cells after 24 h of treatment (Figure 3A). Thereafter, we added intermediate concentration between 0.5 and 1 µM of Pyri to confirm these changes in detail. Acridine orange-stained cells were also analyzed by flow cytometer and the data indicated that as concentrations of Pyri increased, percentage of acridine orange-stained cells was also significantly increased especially at 24 h treatment (Figure 3B). As LC3 has reported to play an important role in autophagosome formation and maturation [60], hence Pyri-induced LC3 expression was analyzed by immunocytochemistry. As depicted in the third panel of Figure 3A, LC3 was substantially expressed at 1 µM concentration of Pyri at 24h, and this was similar to the results obtained in AO and MDC staining assays. On the contrary, based on TUNEL assay, apoptosis activation was mostly observed at 1 µM of Pyri following 48 h of treatment (Figure 3A). These results suggested that Pyri can induce both autophagy and apoptosis, but these processes may not be induced simultaneously by the drug.

### 2.6. Pyri Increased the Expression of Autophagy Markers Including LC3II in KBM5 Cells

As LC3II has been reported to play an important role in autophagosome formation and maturation [60], the expression levels of LC3II and other related markers like Atg7 and Beclin-1 were analyzed. Activation of Atg7 and phospho-Beclin-1 is an essential regulator of autophagosome assembly, and hence we evaluated influence of Pyri on the possible expression of LC3II, Atg7, p-Beclin-1 and Beclin-1 by Western blot analysis. As depicted in Figure 3C, expression of LC3II, Atg7, p-Beclin-1 and Beclin-1 were substantially increased with increasing concentration of Pyri. On the contrary, the changes in the expression levels of previously observed autophagy markers did not directly correlate with the increasing time intervals. The results clearly suggested that LC3II, Atg7, p-Beclin-1 and Beclin-1 levels were maximally induced after 24 h treatment with Pyri (1 µM) (Figure 3D). 

### 2.7. Pyri Induced Cell Death by Alternative Pathways upon Autophagy Inhibition

**Figure 4:**
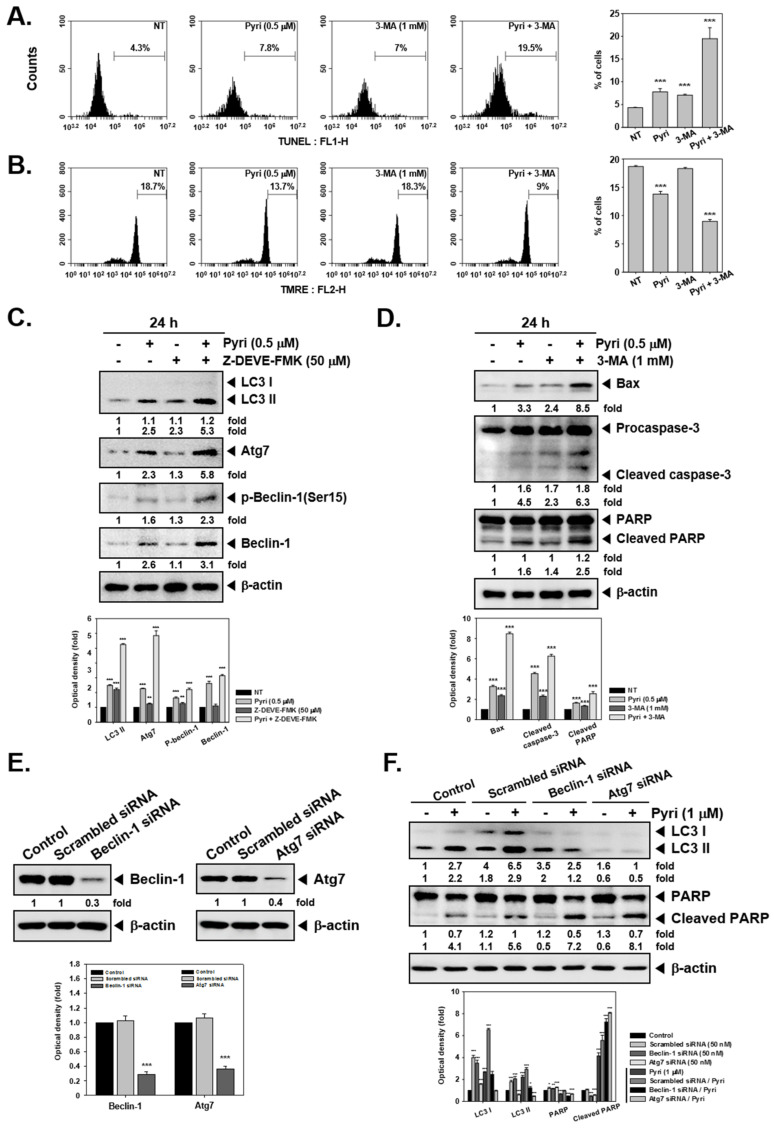
Inhibition of autophagy can sensitize the tumor cells to Pyri-induced apoptotic cell death. (**A**,**B**) KBM5 cells (1 × 10^6^ cells/well) were pretreated with 1 mM concentrations of 3-MA for 30 min, and then exposed to Pyri (0.5 μM) for 24 h. Then, the cells were fixed and incubated using TUNEL reaction solution or TMRE and then analyzed by a flow cytometry. (**C**,**D**) KBM5 cells (1 × 10^6^ cells/well) were pretreated with 50 μM of Z-DEVE-FMK and 1 mM concentrations of 3-MA for 30 min, then exposed to Pyri (0.5 μM) for 24 h. Thereafter the western blot analysis was carried out. (**E**,**F**) The effects of genetic inhibition of autophagy by knockdown of Beclin-1 or Atg7 on Pyri-mediated apoptosis. Cells were treated with 1 µM of Pyri for 24h, then LC3 and cleaved PARP levels were examined by western blot. *** *p* < 0.001 vs. non-treated (NT) cells, ** *p* < 0.01 vs. non-treated (NT) cells, * *p* < 0.05 vs. non-treated (NT) cells. The results shown are representative of three independent experiments.

We next evaluated the alterations in Pyri-induced cell death upon blockade of autophagy. For this, autophagy inhibitors 3-Methyladenine (3-MA) was used. As our above findings indicated that Pyri-induced apoptosis and autophagy occurred at different time intervals, the correlation between Pyri-induced apoptosis and autophagy was analyzed. We noted that 1 mM 3-MA inhibited the cell viability with Pyri. Interestingly, treatment of Pyri on cells which was pre-treated with 3-MA for 30 min exhibited a decrease in the cell viability as compared to pyri alone treated cells (Figure 3E). Overall, the results suggested that the apoptosis was induced upon Pyri treatment even when autophagy was inhibited effectively in KBM-5 cells. We also observed the extent of apoptosis in 3-MA and Pyri treated cells by TUNEL assay. As shown in results, both Pyri and 3-MA alone slightly increased the apoptosis, but in co-treated cells, apoptosis was induced to a substantial extent as compared to single treatment regimen (Figure 4A). Then cells were also subjected to mitochondrial membrane potential (MMP) assay, where also single treatment of Pyri and 3-MA showed slightly change but in co-treated cells, loss of MMP was substantially induced by both Pyri and 3-MA in combination (Figure 4B). Next, the autophagy and apoptosis markers were monitored by Western blot analysis. We observed that Pyri increased the levels of both autophagy and apoptosis markers and caspase inhibitor Z-DEVE-FMK treatment increased the expression of autophagy-related markers. On the other hand, autophagy inhibitor 3-MA increased apoptosis by causing an activation of PARP and caspase-3 cleavage (Figure 4C,D). Interestingly, 3-MA and Pyri combination exhibited pronounced effects on the caspase-3 cleavage. The untagged bands shown in the blot are non-specific bands.

### 2.8. Pyri Induced LC3II Expression through Causing Beclin-1 and Atg7 Activatio

As Beclin-1, Atg7, and LC3 activation has been well established in autophagy, the possible effects of Beclin-1 and Atg7 depletion upon sirNA transfection was analyzed by western blotting. The cells were transfected with siRNA to knockdown the Beclin-1 and Atg7 and treated with Pyri (1 µM) for 24 h. Beclin-1 and Atg7 siRNA transfected showed a remarkable decrease in LC3II expression, even upon Pyri treatment. However, autophagy gene silencing promoted substantial PARP cleavage as compared to that in control and scrambled siRNA transfected cells (Figure 4E,F).

### 2.9. Pyri Suppressed STAT5 Phosphorylation in KBM5 Cells

**Figure 5:**
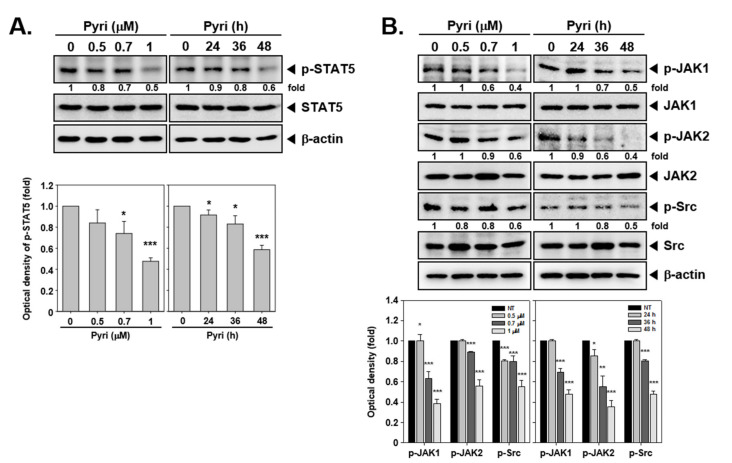

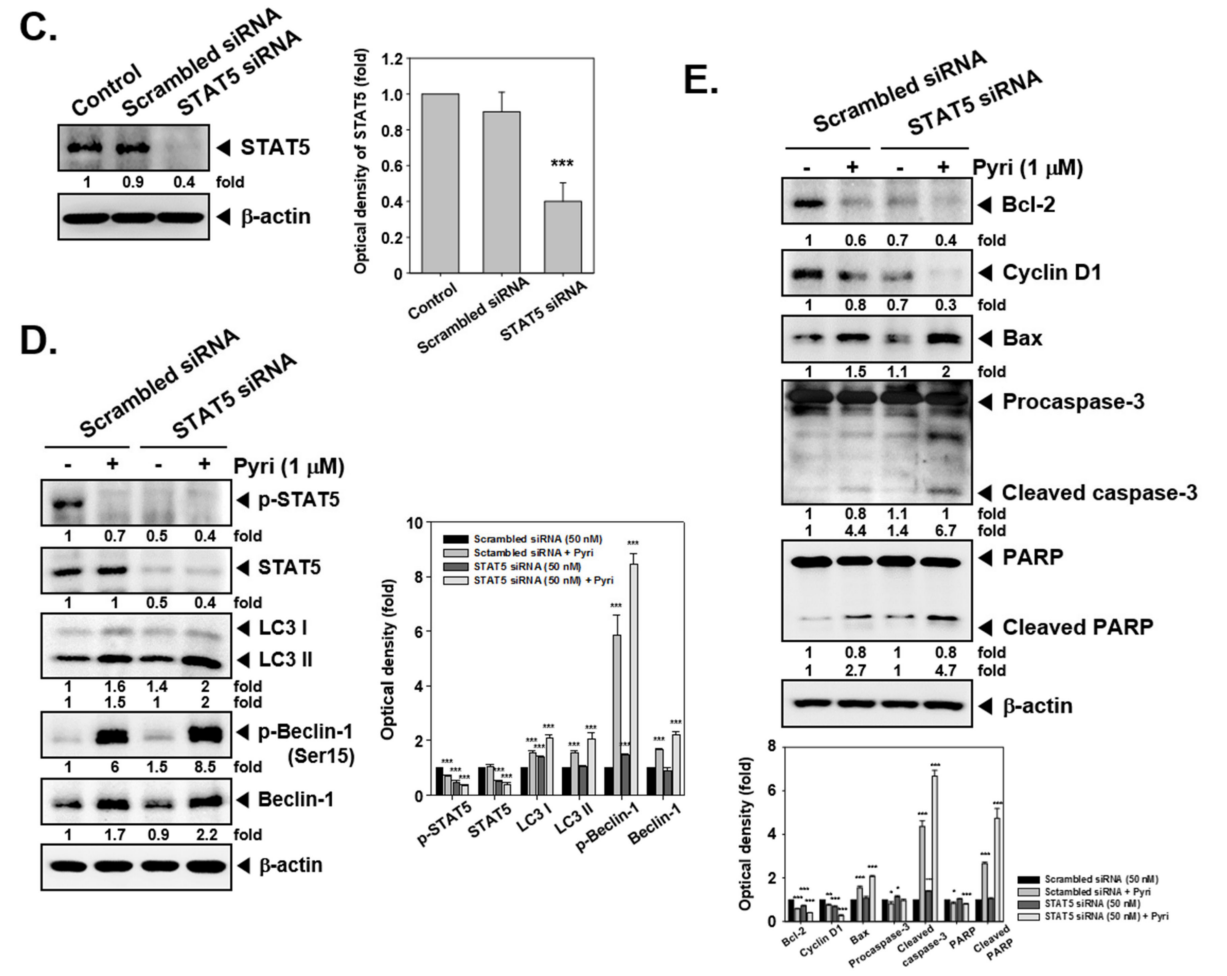
Inactivation of STAT5/Bcl-2 signaling can contribute to Pyri-induced cell death. (**A**,**B**) Pyri treatment repressed the expression of p-STAT5, p-JAK1, p-JAK2 and p-Src, as assayed by western blot. Concentrations dependent manners (*left*), time-dependent manners (*right*). (**C**–**E**) Western blotting was used to assess p-STAT5, STAT5 targets (Bcl-2 and cyclin D1), apoptosis-related proteins, and autophagy-related proteins in STAT5 siRNA or scrambled siRNA-transfected KBM5 cells treated with Pyri. *** *p* < 0.001 vs. non-treated (NT) cells, ** *p* < 0.01 vs. non-treated (NT) cells, * *p* < 0.05 vs. non-treated (NT) cells. The results shown are representative of three independent experiments.

As STAT5 activation plays an important role in cancer progression, the impact of Pyri on STAT5 activation was evaluated. As shown on Figure 5A, concentrations dependent manners (*left*) and time-dependent manners (*right*), Pyri substantially suppressed phosphorylation of STAT5 in a concentration and time-dependent fashion without affecting total STAT5 expression. 

### 2.10. Pyri Down-Regulated the STAT5 Upstream Signals JAK1/2, and Src Activation

JAK1, JAK2, and Src can function as upstream signals for STAT5 activation, so we determined whether Pyri can down-regulate the activation of JAK1, JAK2, and Src kinases. The results showed that Pyri down-regulated the activation of JAK1, JAK2 and Src by effectively suppressing their phosphorylation (Figure 5B), but no effect was noted on total JAK1, JAK2, and Src protein expression. Concentrations dependent manners (*left*), time-dependent manners (*right*).

### 2.11. Pyri Induced Apoptosis and Autophagy in STAT5 Deleted Cells

To confirm the relationship between STAT5 activation, apoptosis, autophagy activation, STAT5 was knocked down in KBM5 cells by STAT5 siRNA transfection, and then treated with Pyri-for 24 h. STAT5 siRNA blocked the STAT5 expression (Figure 5C), but was found to increase autophagy and apoptosis activation through promoting an increase in the levels of autophagic and apoptotic markers concomitant with the suppression of anti-apoptotic proteins (Figure 5D,E). The untagged bands shown in the blot are non-specific bands.

### 2.12. Bcl-2 Overexpression Induced the Autophagy Activation in KBM5 Cells

**Figure 6:**
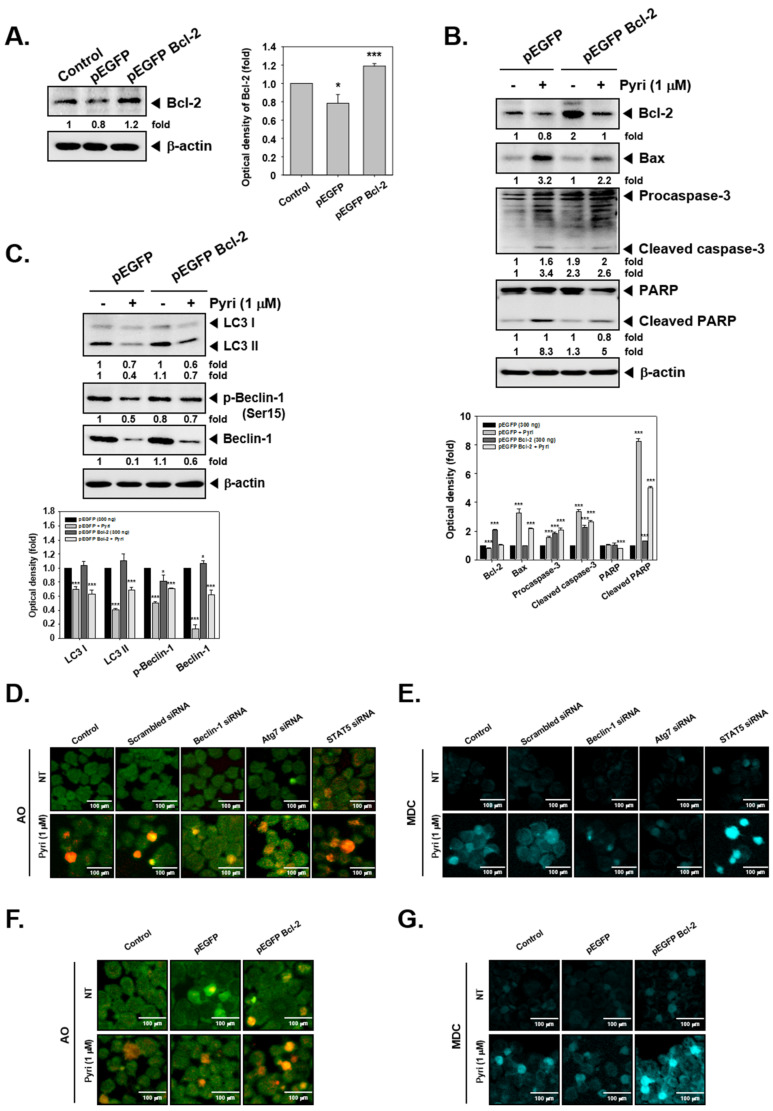
Pyri-induced cell death following Beclin, Atg7, STAT5, and Bcl-2 interference. (**A**–**C**) The possible effects of genetic overexpression of Bcl-2 on Pyri-mediated apoptosis and autophagy. The cells were treated with 1 µM of Pyri for 24h, and then western blotting was performed. *** *p* < 0.001 vs. non-treated (NT) cells, * *p* < 0.05 vs. non-treated (NT) cells. (**D**,**E**) KBM5 cells (2 × 10^7^ cells/well) were transfected with beclin-1, Atg7, and STAT5 siRNA for 48 h. Then treated with Pyri (1 µM) for 24 h and analyzed by AO and MDC staining. (**F**,**G**) Bcl-2 was over expressed by pEGFP-Bcl-2 DNA. Then the cells were stained by AO and MDC. The results shown are representative of three independent experiments.

To confirm the correlation between anti-apoptotic protein expression and autophagy activation, *bcl-2* gene was overexpressed by pEGFP bcl-2 transfection. pEGFP bcl-2 transfection increased the bcl-2 expression (Figure 6A), but reduced the apoptosis activation through affecting PARP and caspase-3 cleavage (Figure 6B). The untagged bands shown in the blot are non-specific bands. However, overexpression of apoptotic protein Bcl-2 2 increased autophagy activation through causing an up-regulation of LC3II, phospho-Beclin-1, and Beclin-1 expression upon Pyri treatment (Figure 6C).

### 2.13. Effect on Induction of Autophagy by Pyri after Beclin-1, Atg7, STAT5, and Bcl-2 Gene Transfection

We next transfected the genes Beclin-1, Atg7, STAT5, and Bcl-2 related to autophagy and observed changes in autophagy by pyri. First, we transfected KBM5 cells with Beclin-1, Atg7, and STAT5 siRNA. Thereafter, the transfected cells were treated with Pyri (1 µM), and autophagy was evaluated by AO and MDC assays. Figure 6D shown that knockdown of beclin-1 and Atg7 reduced the pyri-induced AO staining with autophagy-produced acidic vesicular organelles. Similarly, beclin-1 and Atg7 transfected cells showed that autophagy-produced acidic vesicular organelles were decreased in staining by mdc was observed (Figure 6E). On the other hand, STAT5 gene knockdown showed staining of greater number of autophagy-producing acid vesicle organelles. Then we overexpressed the Bcl-2 gene in KBM5 cells by pEGFP Bcl-2 DNA transfection. Bcl-2 overexpression increased the autophagy-producing acid vesicle organelles staining as observed by AO and MDC (Figure 6F,G). These results suggest that the knockdown of beclin-1 and Atg7 genes, which are related to autophagy, can reduce the staining of acid vesicle organelles, whereas knockdown of STAT5 increases autophagy. In addition, it was shown that overexpression of Bcl-2 increased autophagy as opposed to the decrease of apoptosis.

## 3. Discussion

In this study, we determined the influence of Pyri on different forms of cell death and STAT5 activation in KBM5 cells. We also studied the differential effects of Pyri on autophagy and apoptosis and its impact on the expression of various gene signatures regulating these two important processes.

Because the importance of apoptosis in tumor elimination, first, we investigated the effects of Pyri on apoptosis activations in KBM5 cells [61]. Apoptosis is one of the important process to remove the mutated and injured cells and thereby preventing their oncogenic transformation [62,63,64,65]. In the apoptosis process, cell death can be potentially mediated by several important proteins such as caspase 3, 8, 9 and PARP [66,67,68]. However, a number of tumorigenic proteins like Bcl family, Mcl-1, survivin, IAP-1/2, COX-2, cyclin D1, VEGF, and MMP-9 can negate the apoptotic influence and promote tumor cell survival [69,70,71,72,73,74,75,76,77]. We analyzed the Pyri-induced apoptotic cell death by cell cycle, Annexin V, TUNEL and live and dead assays. The ratio of dead cells ratio was observed to increase and the expression level of activated apoptotic proteins (caspase-3, 8, 9, and PARP) was also enhanced upon exposure to Pyri. Additionally, the impact of Pyri on cell proliferation was analyzed in several CML cells the data indicated that Pyri suppressed cellular growth by promoting activation of apoptotic machinery within the tumor cells. [78].

Autophagy is another important process to prevent the normal functioning of cells and organs from getting mutated or disrupted [42,43]. LC3 plays a key role in the assembly of autophagosome produce by conjugation of LC3Ⅰ and LC3Ⅱ [79,80,81]. Atg7 and Atg5 are well-known autophagy-related proteins, which can promote autophagy activation and eliminate the damaged cytoplasmic organelles and cells from the body [82,83,84]. Thus, both LC3 and ATG7 were used as representative autophagosomal markers in this study. We observed a substantial autophagy activation in a time and concentration dependent manner upon exposure to Pyri. However, the extent of apoptosis activation, was established to be independent of time but still correlated with increasing drug concentration. Moreover, as autophagic activity was increased, the levels of autophagosome production and autophagy-related markers were also simultaneously found to be augmented. 

We also investigated the possible cross-talk between apoptosis and autophagy activation induced by Pyri. In our experiments, Z-DEVE-FMK induced the autophagosomal markers like LC3Ⅱ, Atg7, and beclin-1. And 3-MA dramatically increased the apoptosis activation with caspase-3 and PARP cleavage. In addition, beclin-1 and Atg7 gene silencing along with Pyri treatment exerted a substantial effect on PARP cleavage. These results suggested that Pyri-induced autophagy and apoptosis activity were inversely correlated with specific molecular mechanisms

Uncontrolled STAT5 can act as a tumor-promoting factor and drive tumor growth and metastasis [48,49,85]. The Western blot data indicated that Pyri treatment promoted STAT5 and upstream signals (JAK1/2, Src) activation in KBM5 cells in a concentration and time-dependent fashions. Interestingly, STAT5 gene silencing with Pyri markedly increased the expression of both the apoptotic and autophagic markers, thereby suggesting that the drug can regulate different forms of cell death via modulating STAT5 phosphorylation.

In conclusion, this study suggested that Pyri predominantly induced complementary interactions between apoptosis and autophagy activation in tumor cells. Additionally, it can increase the apoptosis and autophagy activation effectively by causing effective suppression of oncogenic transcription factor STAT5. Therefore, Pyri may have a potential role in cancer prevention and treatment by affecting multiple tumor-promoting mechanism(s).

## 4. Materials and Methods

### 4.1. Reagents

Pyrimethamine (pyri, Figure 1A) was purchased from Sigma-Aldrich (St. Louis, Missouri, USA). pyri stock solution (10 mM) was prepared in, storage at −20 °C and finally diluted in cell culture medium to use. Fetal bovine serum (FBS), and penicillin-streptomycin mixture were purchased from Thermo Fisher Scientific Inc (Waltham, Massachusetts, USA). 3-(4,5-dimethylthiazol-2-yl)-2,5-diphenyltetrazolium bromide (MTT), Tris base, glycine, NaCl, sodium dodecylsulfate (SDS), bovine serum albumin (BSA), and autofluorescent agent monodansylcadaverine (MDC) were purchased from Sigma-Aldrich (St. Louis, Missouri, USA). Alexa Fluor^®^ 594 donkey anti-rabbit IgG (H+L) antibody was obtained from Life Technologies (Carlsbad, California, USA). Anti-LC3, anti-Beclin-1, anti-phospho-Beclin-1, anti-Atg7, anti-cyclin D1, anti-phospho-STAT5, anti-phospho-JAK1(Tyr1022/1023), anti-JAK1, anti-phospho-JAK2(Tyr1007/1008), anti-JAK2, anti-phospho-Src(Tyr416), anti-caspase-9, anti-cleaved-caspase-8, and anti-cleaved-caspase-3 antibodies were purchased from Cell Signaling Technology (Danvers, Massachusetts, USA). Anti-Bax, anti-phospho-p53, anti-p53, anti-mcl-1, anti-STAT5, anti-Bcl-2, anti-Bcl-xL, anti-Survivin, anti-IAP-1, anti-IAP-2, anti-COX-2, anti-VEGF, anti-MMP-9 (matrix metalloproteinase-9), anti-caspase-8, anti-caspase-3, anti-PARP, and anti-β-actin antibodies were purchased from Santa Cruz Biotechnology (Dallas, Texas, USA). TUNEL (terminal transferase-mediated dUTP-fluorescein nick end labeling) assay kit was from Roche Diagnostics GmbH (Mannheim, Baden-Württemberg, Germany). Acridin Orange was purchased from Immuno chemistry technology (Bloomington, Indiana, USA). FITC Annexin V Apoptosis Detection Kit I was purchased from BD Biosciences (Franklin Lakes, New Jersey, USA).

### 4.2. Cell Lines and Culture Conditions

Chronic myelogenous leukemia (CML) cell lines KBM5, THP-1, K562, KCL22, and LAMA84 cells were obtained from American Type Culture Collection (Manassas, VA). KBM5 cells were cultured in IMDM medium containing 10% FBS with 1% penicillin/streptomycin. K562, KCL22, and LAMA84 cells were cultured in RPMI 1640 medium containing 10% FBS with 1% penicillin/streptomycin. Cells were maintained at 37 °C in 5% CO_2_ conditions incubator. 

### 4.3. MTT Assay

KBM5, THP-1, K562, KCL22, and LAMA84 cells (2.5 × 10^4^ cells/well) were treated with pyri (0, 0.5, 1 µM) for indicated time intervals. After pyri treatment, cell viability was measured by MTT assay. MTT solution (2 mg/mL) 30 µL was added into each well for 2 h and 100 µL MTT lysis buffer was added for overnight incubation. The cell viability was measured by VARIOSKAN LUX (Thermo Fisher Scientific Inc, Waltham, Massachusetts, USA) at 570 nm as described previously [86]. 

### 4.4. Western Blot Analysis

KBM5 cells were treated with Pyri with indicated concentrations (0, 0.5, 0.7, 1 µM) and time intervals. Thereafter western blot assay was carried out as elaborated previously [87]. After KBM5 cells were treated with indicated various concentrations and time conditions, cells were harvested and whole cell lysates were obtained. Concentrations of proteins were measured by Bradford reagent (Bio-Rad, Hercules, California, USA) then prepared with equal amounts. Proteins were separated on sodium dodecyl-polyacrylamide gel electrophoresis (SDS-PAGE) and electro-transferred to nitrocellulose membrane. Membranes were blocked by 3 or 5% skim milk in 1×TBST (1×TBS with 0.1% Tween 20) for 1 h at room temperature then probed with primary antibodies at 4 °C for overnight. After membranes were washed by 1×TBST, probed with secondary antibodies horseradish peroxidase (HRP) conjugated anti-rabbit IgG antibodies and anti-mouse IgG antibodies at room temperature for 2 h. Expression levels of proteins were detected by enhanced chemiluminescence (ECL) kit (EZ-Western Lumi Femto, DOGEN, Seoul, Korea). After detection, membranes were stripped for 1 h, and probed with anti-β-actin antibodies as loading control. 

### 4.5. RT-PCR

To confirm the mRNA expression levels of various tumorigenic genes upon Pyri treatment, KBM5 cells were treated with 1 µM of Pyri. Cells were suspended with Trizol, then added chloroform, Iso-propanol to extract the RNA. Extracted RNA were reverse transcribed into cDNA, then reverse transcription polymerase chain reaction (RT-PCR) was performed. Each mRNA was performed with different temperature conditions: Bcl-2 reaction was performed at 94 °C for 15 s, 58 °C for 30 s, 72 °C for 1 min with 28 cycles and extension at 72 °C for 5 min. Bcl-xl reaction was performed at 94 °C for 30 s, 57 °C for 30 s, 72 °C for 1 min with 30 cycles and extension at 72 °C for 7 min. Survivin reaction was performed at 94 °C for 30 s, 55 °C for 30 s, 72 °C for 30 °C with 30 cycles and extension at 72 °C for 7 min. Cyclin D1 reaction was performed at 95 °C for 30 s, 60 °C for 30 s, 72 °C for 30 s with 35 cycles and extension at 72 °C for 10 min. All PCR products were separated on 1% agarose gel with Loading Star (Dynebio, Seongnam, Korea) and bands were detected by UV light. Glyceraldehyde-3-phosphate dehydrogenase (GAPDH) was used as loading control as described earlier [88].

### 4.6. Live and Dead Assay

Pyri-induced cell death was detected using live and dead assay kit based on the procedure elaborated previously [89]. KBM5 cells were treated with Pyri (1 µM) for indicated time conditions (0, 12, 18, 24, 48 h), then cells were stained with 5 µM of Calcein AM and Ethd-1(Ethidium homodimer-1) at 37 °C for 30 min. Cells were attached on slide glass by cytospin and detected by Olympus FluoView FV1000 confocal microscope (scale bar: 100 µm) (Tokyo, Japan). Because live cells have intracellular esterase activity, Calcein AM was disaggregated so cells were detected with green color. But dead cells were damaged on membrane, so Ethd-1 can invade into the cell and combine with nucleic acid and cells can be detected with red color.

### 4.7. Acridine Orange Assay

Pyri-induced autophagy activation was measured with acridine orange staining. Autophagy activation can produce vesicular organelles, and acidic components can stain with acridine orange. Pyri treated KBM5 cells were stained with acridine orange (3.8 µM) for 20 min at 37 °C and attached on slide glass. Cells were detected by Olympus FluoView FV1000 confocal microscope (scale bar: 100 µm) (Tokyo, Japan) and BD Accuri™ C6 Plus Flow Cytometer (BD Biosciences, Becton-Dickinson, Franklin Lakes, NJ, USA) with BD Accuri C6 Plus software.

### 4.8. MDC Staining

Pyri-trated cells were stained with Monodansylcadaverine (MDC) to evaluate autophagy activation through monitoring an increase of acid vesicular organelles. Time and concentration dependent actions of Pyri treated cells were analyzed upon staining with MDC (50 µM) for 20 min at 37 °C. Tells were detected by Olympus FluoView FV1000 confocal microscope (Tokyo, Japan).

### 4.9. Cell Cycle Analysis

KBM5 cells (1 × 10^6^ cells/well) were treated with Pyri (1 µM) for 0, 12, 18, 24, 48 h and washed with 1× PBS. Cells were fixed with cold 70% EtOH and incubated overnight at 4 °C. Then fixed cells were washed with 1× PBS again and incubated with 1 mg/mL RNase A for 1 h at 37 °C. After 1 h, cells were stained with propidium iodide (PI) and analyzed by BD Accuri™ C6 Plus Flow Cytometer (BD Biosciences, Becton-Dickinson, Franklin Lakes, NJ) with BD Accuri C6 Plus software.

### 4.10. Annexin V Assay

KBM5 cells (1 × 10^6^ cells/well) were treated with Pyri (1 µM) for 0, 12, 18, 24, 48 h and washed with 1× PBS. Cells were collected and resuspended in fresh 1× PBS with FITC tagged Annexin V antibody and PI staining for 15 min at room temperature. Apoptosis of cells was analyzed by BD Accuri™ C6 Plus Flow Cytometer (BD Biosciences, Becton-Dickinson, Franklin Lakes, NJ) with BD Accuri C6 Plus software 

### 4.11. TUNEL Assay

Pyri-induced apoptotic changes were analyzed by TUNEL assay as reported previously [90]. KBM5 cells were treated with Pyri (0, 0.5, 1 µM) for 0, 12, 18, 24 and 48 h. Cells were probed with TUNEL enzyme and label 1 h at 37 °C. Cells were placed on slide glass using by cytospin and detected by Olympus FluoView FV1000 confocal microscope (scale bar: 100 µm) (Tokyo, Japan) and BD Accuri™ C6 Plus Flow Cytometer (BD Biosciences, Becton-Dickinson, Franklin Lakes, NJ) with BD Accuri C6 Plus software.

### 4.12. Mitochondrial Membrane Potential (MMP) Assay

The cells were treated with Pyri (1 µM) and 3-MA (1 mM) for 24 h, then harvested to PBS washing. Thereafter they were stained by tetramethylrhodamine ethyl ester (TMRE) with 500 µL PBS in final concentration 50 nM. The cells were then incubated for 30 min, and then analyzed by BD AccuriTM C6 Plus Flow Cytometer (BD Biosciences, Becton-Dickinson, Franklin Lakes, NJ).

### 4.13. Immunocytochemistry for LC3 Expression

LC3 expression with Pyri (1 µM) for 24 and 48 h was also analyzed using immunocytochemistry as elaborated before. KBM5 cells were fixed with 4% paraformaldehyde (PFA) in 1× PBS for 20 min at room temperature and permeabilised by 0.2% triton X-100 for 10 min. Cells were blocked with 5% BSA in 1× PBS for 1 h, then probed with anti-LC3 antibody at 4 °C for overnight. The next day, cells were washed with 1× PBS and probed with secondary antibodies Alexa Fluor^®^ 488 donkey anti-rabbit IgG (H+L) for 1 h. Nuclei was stained with DAPI (1 µg/mL) for 3 min and cells were mounted by Fluorescent Mounting Medium (Golden Bridge International Labs, Mukilteo, WA). Thereafter cells were visualized by fluorescence microscope Olympus FluoView FV1000 confocal microscope (scale bar: 100 µm) (Tokyo, Japan) as described earlier [91].

### 4.14. Knockdown of Beclin-1 and Atg7 by siRNA Transfection

KBM5 cells were transfected with 50 nM of Beclin-1, Atg7, and scrambled siRNA for 48 h in penicillin/streptomycin-free IMDM media using by Neon™ Transfection System (Invitrogen, Carlsbad, CA). After transfection, the cells were treated with Pyri (1 µM) for 24 h in complete media and analyzed by acridine orange staining. Scrambled siRNA was used as positive control.

### 4.15. Blockage of STAT5 Expression by siRNA

STAT5 expression was knocked down by using STAT5 siRNA. KBM5 cells were resuspended with 50 nM of STAT5 or scrambled siRNA in 120 μL of Neon Resuspension Buffer R then transfected by Neon™ Transfection System (Invitrogen, Carlsbad, CA) for 36 h in penicillin/streptomycin-free IMDM media. The cells were then treated with Pyri (1 µM) for 24 h for additional experiments.

### 4.16. Bcl-2 Overexpression Studies

For overexpression of Bcl-2, KBM5 cells were transfected with pEGFP Bcl-2 or pEGFP DNA (provided by Case Western Reserve University). Both DNA were transfected with 2000 ng in penicillin/streptomycin-free IMDM media using Neon™ Transfection System (Invitrogen, Carlsbad, CA) for 24 h. Thereafter the cells were exposed to Pyri (1 µM) for 24 h in complete media, and the whole cell extracts were prepared for Western blot analysis.

### 4.17. Statistical Analysis

All numerical values are represented as the mean ± SD. The statistical significance of the data compared with the untreated control was determined using the Student’s unpaired *t*-test. The significance was set at *p < 0.05, **p < 0.01, and ***p < 0.001.

## Data Availability

Not applicable.

## References

[1] Montoya J.G., Liesenfeld O. (2004). Toxoplasmosis. Lancet.

[2] USPHS/IDSA Prevention of Opportunistic Infections Working Group (2000). 1999 USPHS/IDSA guidelines for the prevention of opportunistic infections in persons infected with human immunodeficiency virus. Clin. Infect. Dis..

[3] Bygbjerg I.C. (1985). Pyrimethamine-induced alterations in human lymphocytes in vitro. Mechanisms and reversal of the effect. Acta Pathol. Microbiol. Immunol. Scand. C.

[4] Bygbjerg I.C., Odum N., Theander T.G. (1986). Effect of pyrimethamine and sulphadoxine on human lymphocyte proliferation. Trans. R Soc. Trop. Med. Hyg..

[5] Viora M., De Luca A., D’Ambrosio A., Antinori A., Ortona E. (1996). In vitro and in vivo immunomodulatory effects of anti-Pneumocystis carinii drugs. Antimicrob. Agents Chemother..

[6] Van der Werff Ten Bosch J., Schotte P., Ferster A., Azzi N., Boehler T., Laurey G., Arola M., Demanet C., Beyaert R., Thielemans K. (2002). Reversion of autoimmune lymphoproliferative syndrome with an antimalarial drug: Preliminary results of a clinical cohort study and molecular observations. Br. J. Haematol..

[7] Pierdominici M., Giammarioli A.M., Gambardella L., De Felice M., Quinti I., Iacobini M., Carbonari M., Malorni W., Giovannetti A. (2005). Pyrimethamine (2,4-diamino-5-p-chlorophenyl-6-ethylpyrimidine) induces apoptosis of freshly isolated human T lymphocytes, bypassing CD95/Fas molecule but involving its intrinsic pathway. J. Pharmacol. Exp. Ther..

[8] Calabretta B., Perrotti D. (2004). The biology of CML blast crisis. Blood.

[9] Jung Y.Y., Shanmugam M.K., Chinnathambi A., Alharbi S.A., Shair O.H.M., Um J.Y., Sethi G., Ahn K.S. (2019). Fangchinoline, a Bisbenzylisoquinoline Alkaloid can Modulate Cytokine-Impelled Apoptosis via the Dual Regulation of NF-kappaB and AP-1 Pathways. Molecules.

[10] Kim C., Lee J.H., Kim S.H., Sethi G., Ahn K.S. (2015). Artesunate suppresses tumor growth and induces apoptosis through the modulation of multiple oncogenic cascades in a chronic myeloid leukemia xenograft mouse model. Oncotarget.

[11] Ahn K.S., Sethi G., Aggarwal B.B. (2008). Reversal of chemoresistance and enhancement of apoptosis by statins through down-regulation of the NF-kappaB pathway. Biochem. Pharmacol..

[12] Shishodia S., Sethi G., Ahn K.S., Aggarwal B.B. (2007). Guggulsterone inhibits tumor cell proliferation, induces S-phase arrest, and promotes apoptosis through activation of c-Jun N-terminal kinase, suppression of Akt pathway, and downregulation of antiapoptotic gene products. Biochem. Pharmacol..

[13] Danial N.N., Rothman P. (2000). JAK-STAT signaling activated by Abl oncogenes. Oncogene.

[14] Jain S.K., Langdon W.Y., Varticovski L. (1997). Tyrosine phosphorylation of p120cbl in BCR/abl transformed hematopoietic cells mediates enhanced association with phosphatidylinositol 3-kinase. Oncogene.

[15] Mizuchi D., Kurosu T., Kida A., Jin Z.H., Jin A., Arai A., Miura O. (2005). BCR/ABL activates Rap1 and B-Raf to stimulate the MEK/Erk signaling pathway in hematopoietic cells. Biochem. Biophys Res. Commun..

[16] Arora L., Kumar A.P., Arfuso F., Chng W.J., Sethi G. (2018). The Role of Signal Transducer and Activator of Transcription 3 (STAT3) and Its Targeted Inhibition in Hematological Malignancies. Cancers.

[17] Ong P.S., Wang L.Z., Dai X., Tseng S.H., Loo S.J., Sethi G. (2016). Judicious Toggling of mTOR Activity to Combat Insulin Resistance and Cancer: Current Evidence and Perspectives. Front. Pharmacol..

[18] Kantarjian H.M., Talpaz M., Giles F., O’Brien S., Cortes J. (2006). New insights into the pathophysiology of chronic myeloid leukemia and imatinib resistance. Ann. Intern. Med..

[19] Hsieh Y.S., Yang S.F., Sethi G., Hu D.N. (2015). Natural bioactives in cancer treatment and prevention. Biomed. Res. Int..

[20] Shanmugam M.K., Warrier S., Kumar A.P., Sethi G., Arfuso F. (2017). Potential Role of Natural Compounds as Anti-Angiogenic Agents in Cancer. Curr. Vasc. Pharmacol..

[21] Yang S.F., Weng C.J., Sethi G., Hu D.N. (2013). Natural bioactives and phytochemicals serve in cancer treatment and prevention. Evid. Based Complement. Alternat. Med..

[22] Dai X., Zhang J., Arfuso F., Chinnathambi A., Zayed M.E., Alharbi S.A., Kumar A.P., Ahn K.S., Sethi G. (2015). Targeting TNF-related apoptosis-inducing ligand (TRAIL) receptor by natural products as a potential therapeutic approach for cancer therapy. Exp. Biol. Med..

[23] Shanmugam M.K., Ong T.H., Kumar A.P., Lun C.K., Ho P.C., Wong P.T., Hui K.M., Sethi G. (2012). Ursolic acid inhibits the initiation, progression of prostate cancer and prolongs the survival of TRAMP mice by modulating pro-inflammatory pathways. PLoS ONE.

[24] Aggarwal V., Tuli H.S., Varol A., Thakral F., Yerer M.B., Sak K., Varol M., Jain A., Khan M.A., Sethi G. (2019). Role of Reactive Oxygen Species in Cancer Progression: Molecular Mechanisms and Recent Advancements. Biomolecules.

[25] Kim C., Lee J.H., Ko J.H., Chinnathambi A., Alharbi S.A., Shair O.H.M., Sethi G., Ahn K.S. (2019). Formononetin Regulates Multiple Oncogenic Signaling Cascades and Enhances Sensitivity to Bortezomib in a Multiple Myeloma Mouse Model. Biomolecules.

[26] Hwang S.T., Kim C., Lee J.H., Chinnathambi A., Alharbi S.A., Shair O.H.M., Sethi G., Ahn K.S. (2019). Cycloastragenol can negate constitutive STAT3 activation and promote paclitaxel-induced apoptosis in human gastric cancer cells. Phytomedicine.

[27] Kerr J.F., Wyllie A.H., Currie A.R. (1972). Apoptosis: A basic biological phenomenon with wide-ranging implications in tissue kinetics. Br. J. Cancer.

[28] Wyllie A.H., Kerr J.F., Currie A.R. (1980). Cell death: The significance of apoptosis. Int. Rev. Cytol..

[29] Cryns V., Yuan J. (1998). Proteases to die for. Genes Dev..

[30] Los M., Wesselborg S., Schulze-Osthoff K. (1999). The role of caspases in development, immunity, and apoptotic signal transduction: Lessons from knockout mice. Immunity.

[31] Cohen G.M. (1997). Caspases: The executioners of apoptosis. Biochem. J..

[32] Lazebnik Y.A., Kaufmann S.H., Desnoyers S., Poirier G.G., Earnshaw W.C. (1994). Cleavage of poly(ADP-ribose) polymerase by a proteinase with properties like ICE. Nature.

[33] Los M., Herr I., Friesen C., Fulda S., Schulze-Osthoff K., Debatin K.M. (1997). Cross-resistance of CD95- and drug-induced apoptosis as a consequence of deficient activation of caspases (ICE/Ced-3 proteases). Blood.

[34] Kaufmann S.H., Desnoyers S., Ottaviano Y., Davidson N.E., Poirier G.G. (1993). Specific proteolytic cleavage of poly(ADP-ribose) polymerase: An early marker of chemotherapy-induced apoptosis. Cancer Res..

[35] Nicholson D.W., Ali A., Thornberry N.A., Vaillancourt J.P., Ding C.K., Gallant M., Gareau Y., Griffin P.R., Labelle M., Lazebnik Y.A. (1995). Identification and inhibition of the ICE/CED-3 protease necessary for mammalian apoptosis. Nature.

[36] Sethi G., Sung B., Kunnumakkara A.B., Aggarwal B.B. (2009). Targeting TNF for Treatment of Cancer and Autoimmunity. Adv. Exp. Med. Biol..

[37] Ahn K.S., Sethi G., Jain A.K., Jaiswal A.K., Aggarwal B.B. (2006). Genetic deletion of NAD(P)H:quinone oxidoreductase 1 abrogates activation of nuclear factor-kappaB, IkappaBalpha kinase, c-Jun N-terminal kinase, Akt, p38, and p44/42 mitogen-activated protein kinases and potentiates apoptosis. J. Biol. Chem..

[38] Jung Y.Y., Lee J.H., Nam D., Narula A.S., Namjoshi O.A., Blough B.E., Um J.Y., Sethi G., Ahn K.S. (2018). Anti-myeloma Effects of Icariin Are Mediated Through the Attenuation of JAK/STAT3-Dependent Signaling Cascade. Front. Pharmacol..

[39] Jung Y.Y., Shanmugam M.K., Narula A.S., Kim C., Lee J.H., Namjoshi O.A., Blough B.E., Sethi G., Ahn K.S. (2019). Oxymatrine Attenuates Tumor Growth and Deactivates STAT5 Signaling in a Lung Cancer Xenograft Model. Cancers.

[40] Deng S., Shanmugam M.K., Kumar A.P., Yap C.T., Sethi G., Bishayee A. (2019). Targeting autophagy using natural compounds for cancer prevention and therapy. Cancer.

[41] Praharaj P.P., Naik P.P., Panigrahi D.P., Bhol C.S., Mahapatra K.K., Patra S., Sethi G., Bhutia S.K. (2019). Intricate role of mitochondrial lipid in mitophagy and mitochondrial apoptosis: Its implication in cancer therapeutics. Cell Mol. Life Sci..

[42] Mizushima N. (2005). The pleiotropic role of autophagy: From protein metabolism to bactericide. Cell Death Differ..

[43] Mizushima N. (2007). Autophagy: Process and function. Genes Dev..

[44] Klionsky D.J. (2007). Autophagy: From phenomenology to molecular understanding in less than a decade. Nat. Rev. Mol. Cell Bio..

[45] Kroemer G., Jaattela M. (2005). Lysosomes and autophagy in cell death control. Nat. Rev. Cancer.

[46] Yun C.W., Lee S.H. (2018). The Roles of Autophagy in Cancer. Int. J. Mol. Sci..

[47] Bellodi C., Lidonnici M.R., Hamilton A., Helgason G.V., Soliera A.R., Ronchetti M., Galavotti S., Young K.W., Selmi T., Yacobi R. (2009). Targeting autophagy potentiates tyrosine kinase inhibitor-induced cell death in Philadelphia chromosome-positive cells, including primary CML stem cells. J. Clin. Invest..

[48] Weber A., Borghouts C., Brendel C., Moriggl R., Delis N., Brill B., Vafaizadeh V., Groner B. (2015). Stat5 Exerts Distinct, Vital Functions in the Cytoplasm and Nucleus of Bcr-Abl(+) K562 and Jak2(V617F)(+) HEL Leukemia Cells. Cancers.

[49] Berger A., Sexl V., Valent P., Moriggl R. (2014). Inhibition of STAT5: A therapeutic option in BCR-ABL1-driven leukemia. Oncotarget.

[50] Kim C., Cho S.K., Kapoor S., Kumar A., Vali S., Abbasi T., Kim S.H., Sethi G., Ahn K.S. (2014). beta-Caryophyllene oxide inhibits constitutive and inducible STAT3 signaling pathway through induction of the SHP-1 protein tyrosine phosphatase. Mol. Carcinog..

[51] Lee J.H., Kim C., Sethi G., Ahn K.S. (2015). Brassinin inhibits STAT3 signaling pathway through modulation of PIAS-3 and SOCS-3 expression and sensitizes human lung cancer xenograft in nude mice to paclitaxel. Oncotarget.

[52] Lee J.H., Chiang S.Y., Nam D., Chung W.S., Lee J., Na Y.S., Sethi G., Ahn K.S. (2014). Capillarisin inhibits constitutive and inducible STAT3 activation through induction of SHP-1 and SHP-2 tyrosine phosphatases. Cancer Lett..

[53] Mohan C.D., Bharathkumar H., Bulusu K.C., Pandey V., Rangappa S., Fuchs J.E., Shanmugam M.K., Dai X., Li F., Deivasigamani A. (2014). Development of a novel azaspirane that targets the Janus kinase-signal transducer and activator of transcription (STAT) pathway in hepatocellular carcinoma in vitro and in vivo. J. Biol. Chem..

[54] Kim C., Lee S.G., Yang W.M., Arfuso F., Um J.Y., Kumar A.P., Bian J., Sethi G., Ahn K.S. (2018). Formononetin-induced oxidative stress abrogates the activation of STAT3/5 signaling axis and suppresses the tumor growth in multiple myeloma preclinical model. Cancer Lett..

[55] Bandapalli O.R., Schuessele S., Kunz J.B., Rausch T., Stutz A.M., Tal N., Geron I., Gershman N., Izraeli S., Eilers J. (2014). The activating STAT5B N642H mutation is a common abnormality in pediatric T-cell acute lymphoblastic leukemia and confers a higher risk of relapse. Haematologica.

[56] Vafaizadeh V., Klemmt P., Brendel C., Weber K., Doebele C., Britt K., Grez M., Fehse B., Desrivieres S., Groner B. (2010). Mammary Epithelial Reconstitution with Gene-Modified Stem Cells Assigns Roles to Stat5 in Luminal Alveolar Cell Fate Decisions, Differentiation, Involution, and Mammary Tumor Formation. Stem Cells.

[57] Gouilleux F., Wakao H., Mundt M., Groner B. (1994). Prolactin induces phosphorylation of Tyr694 of Stat5 (MGF), a prerequisite for DNA binding and induction of transcription. EMBO J..

[58] Thomas S.J., Snowden J.A., Zeidler M.P., Danson S.J. (2015). The role of JAK/STAT signalling in the pathogenesis, prognosis and treatment of solid tumours. Br. J. Cancer.

[59] Shi M., Yang S., Zhu X., Sun D., Sun D., Jiang X., Zhang C., Wang L. (2019). The RAGE/STAT5/autophagy axis regulates senescence in mesangial cells. Cell Signal..

[60] Singh S.S., Vats S., Chia A.Y., Tan T.Z., Deng S., Ong M.S., Arfuso F., Yap C.T., Goh B.C., Sethi G. (2018). Dual role of autophagy in hallmarks of cancer. Oncogene.

[61] Lowe S.W., Lin A.W. (2000). Apoptosis in cancer. Carcinogenesis.

[62] Galluzzi L., Joza N., Tasdemir E., Maiuri M.C., Hengartner M., Abrams J.M., Tavernarakis N., Penninger J., Madeo F., Kroemer G. (2008). No death without life: Vital functions of apoptotic effectors. Cell Death Differ..

[63] Oberst A., Bender C., Green D.R. (2008). Living with death: The evolution of the mitochondrial pathway of apoptosis in animals. Cell Death Differ..

[64] Manu K.A., Shanmugam M.K., Li F., Chen L., Siveen K.S., Ahn K.S., Kumar A.P., Sethi G. (2014). Simvastatin sensitizes human gastric cancer xenograft in nude mice to capecitabine by suppressing nuclear factor-kappa B-regulated gene products. J. Mol. Med..

[65] Sethi G., Shanmugam M.K., Warrier S., Merarchi M., Arfuso F., Kumar A.P., Bishayee A. (2018). Pro-Apoptotic and Anti-Cancer Properties of Diosgenin: A Comprehensive and Critical Review. Nutrients.

[66] Chaitanya G.V., Steven A.J., Babu P.P. (2010). PARP-1 cleavage fragments: Signatures of cell-death proteases in neurodegeneration. Cell Commun. Signal..

[67] Baek S.H., Ko J.H., Lee H., Jung J., Kong M., Lee J.W., Lee J., Chinnathambi A., Zayed M.E., Alharbi S.A. (2016). Resveratrol inhibits STAT3 signaling pathway through the induction of SOCS-1: Role in apoptosis induction and radiosensitization in head and neck tumor cells. Phytomedicine.

[68] Lee J.H., Kim C., Kim S.H., Sethi G., Ahn K.S. (2015). Farnesol inhibits tumor growth and enhances the anticancer effects of bortezomib in multiple myeloma xenograft mouse model through the modulation of STAT3 signaling pathway. Cancer Lett..

[69] Carrington E.M., Zhan Y.F., Brady J.L., Zhang J.G., Sutherland R.M., Anstee N.S., Schenk R.L., Vikstrom I.B., Delconte R.B., Segal D. (2017). Anti-apoptotic proteins BCL-2, MCL-1 and A1 summate collectively to maintain survival of immune cell populations both in vitro and in vivo. Cell Death Differ..

[70] Portt L., Norman G., Clapp C., Greenwood M., Greenwood M.T. (2011). Anti-apoptosis and cell survival: A review. Bba-Mol. Cell Res..

[71] Jaiswal P.K., Goel A., Mittal R.D. (2015). Survivin: A molecular biomarker in cancer. Indian J. Med. Res..

[72] Goradel N.H., Najafi M., Salehi E., Farhood B., Mortezaee K. (2019). Cyclooxygenase-2 in cancer: A review. J. Cell Physiol..

[73] Carmeliet P. (2005). VEGF as a key mediator of angiogenesis in cancer. Oncology-Basel.

[74] Huang H. (2018). Matrix Metalloproteinase-9 (MMP-9) as a Cancer Biomarker and MMP-9 Biosensors: Recent Advances. Sensors.

[75] Qie S., Diehl J.A. (2016). Cyclin D1, cancer progression, and opportunities in cancer treatment. J. Mol. Med..

[76] Kirtonia A., Sethi G., Garg M. (2020). The multifaceted role of reactive oxygen species in tumorigenesis. Cell Mol. Life Sci..

[77] Manu K.A., Shanmugam M.K., Ramachandran L., Li F., Siveen K.S., Chinnathambi A., Zayed M.E., Alharbi S.A., Arfuso F., Kumar A.P. (2015). Isorhamnetin augments the anti-tumor effect of capecitabine through the negative regulation of NF-kappaB signaling cascade in gastric cancer. Cancer Lett..

[78] Wong R.S.Y. (2011). Apoptosis in cancer: From pathogenesis to treatment. J. Exp. Clin. Canc. Res..

[79] Tanida I., Ueno T., Kominami E. (2008). LC3 and Autophagy. Methods Mol. Biol..

[80] Kraft C., Martens S. (2012). Mechanisms and regulation of autophagosome formation. Curr. Opin. Cell Biol..

[81] Yang Z., Klionsky D.J. (2010). Mammalian autophagy: Core molecular machinery and signaling regulation. Curr. Opin. Cell Biol..

[82] Carames B., Hasegawa A., Taniguchi N., Miyaki S., Blanco F.J., Lotz M. (2012). Autophagy activation by rapamycin reduces severity of experimental osteoarthritis. Ann. Rheum Dis..

[83] Bouderlique T., Vuppalapati K.K., Newton P.T., Li L., Barenius B., Chagin A.S. (2016). Targeted deletion of Atg5 in chondrocytes promotes age-related osteoarthritis. Ann. Rheum Dis..

[84] Carames B., Taniguchi N., Otsuki S., Blanco F.J., Lotz M. (2010). Autophagy Is a Protective Mechanism in Normal Cartilage, and Its Aging-Related Loss Is Linked With Cell Death and Osteoarthritis. Arthritis Rheum-Us.

[85] Weber A., Borghouts C., Brendel C., Moriggl R., Delis N., Brill B., Vafaizadeh V., Groner B. (2013). The inhibition of stat5 by a Peptide aptamer ligand specific for the DNA binding domain prevents target gene transactivation and the growth of breast and prostate tumor cells. Pharmaceuticals.

[86] Lee J.H., Kim C., Um J.Y., Sethi G., Ahn K.S. (2019). Casticin-Induced Inhibition of Cell Growth and Survival Are Mediated through the Dual Modulation of Akt/mTOR Signaling Cascade. Cancers.

[87] Li F., Shanmugam M.K., Chen L., Chatterjee S., Basha J., Kumar A.P., Kundu T.K., Sethi G. (2013). Garcinol, a polyisoprenylated benzophenone modulates multiple proinflammatory signaling cascades leading to the suppression of growth and survival of head and neck carcinoma. Cancer Prev. Res..

[88] Yang M.H., Jung S.H., Chinnathambi A., Alahmadi T.A., Alharbi S.A., Sethi G., Ahn K.S. (2019). Attenuation of STAT3 Signaling Cascade by Daidzin Can Enhance the Apoptotic Potential of Bortezomib against Multiple Myeloma. Biomolecules.

[89] Subramaniam A., Loo S.Y., Rajendran P., Manu K.A., Perumal E., Li F., Shanmugam M.K., Siveen K.S., Park J.I., Ahn K.S. (2013). An anthraquinone derivative, emodin sensitizes hepatocellular carcinoma cells to TRAIL induced apoptosis through the induction of death receptors and downregulation of cell survival proteins. Apoptosis.

[90] Ahn K.S., Sethi G., Aggarwal B.B. (2007). Simvastatin potentiates TNF-alpha-induced apoptosis through the down-regulation of NF-kappaB-dependent antiapoptotic gene products: Role of IkappaBalpha kinase and TGF-beta-activated kinase-1. J. Immunol..

[91] Lee J.H., Chinnathambi A., Alharbi S.A., Shair O.H.M., Sethi G., Ahn K.S. (2019). Farnesol abrogates epithelial to mesenchymal transition process through regulating Akt/mTOR pathway. Pharmacol. Res..

